# ‘I could not join because I had to work for pay.’: A qualitative evaluation of *falciparum* malaria pro-active case detection in three rural Cambodian villages

**DOI:** 10.1371/journal.pone.0195809

**Published:** 2018-04-12

**Authors:** Pierluigi Taffon, Gabriele Rossi, Jean-Marie Kindermans, Rafael Van den Bergh, Chea Nguon, Mark Debackere, Lieven Vernaeve, Martin De Smet, Emilie Venables

**Affiliations:** 1 Médecins Sans Frontières, Phnom Penh, Cambodia; 2 Médecins Sans Frontières, Operational Centre Belgium, Brussels, Belgium; 3 Centre for Parasitology, Entomology and Malaria Control, Phnom Penh, Cambodia; 4 Division of Social and Behavioural Sciences, School of Public Health and Family Medicine, University of Cape Town, Cape Town, South Africa; Mahidol-Oxford Tropical Medicine Research Unit, THAILAND

## Abstract

**Background:**

Pro-active case detection (Pro-ACD), in the form of voluntary screening and treatment (VSAT) following community mobilisation about ‘asymptomatic malaria’, is currently being evaluated as a tool for *Plasmodium falciparum* elimination in Preah Vihear Province, Cambodia.

**Methods:**

A qualitative study was conducted to explore community understanding, perceptions, expectations and acceptability of the Pro-ACD intervention in order to identify aspects that could be improved in future Pro-ACD activities. This was ancillary to a three-round VSAT campaign, carried out in three villages between December 2015 and March 2016. Qualitative data collection began shortly after the end of the three rounds of screening. Purposive sampling was used to select participants. Nine focus group discussions with participants (n = 46) and non-participants (n = 40) in the Pro-ACD screening were conducted, in addition to in-depth interviews with key village figures (n = 9).

**Results:**

Health promotion messages were well delivered and received, but it was difficult for many villagers to understand the messages around ‘asymptomatic malaria’. Overall, villagers and village leaders had a positive opinion about the VSAT intervention. Acceptability was high, as a direct consequence of favourable perceptions towards the screening activity: the Pro-ACD intervention was seen by the local population as an effective, inexpensive, reliable and readily available tool to protect individuals and the community from the insurgence of malaria. Physical absence and lack of time (both linked to work-related activities) were the main reasons for non-participation.

**Conclusions:**

Although VSAT was generally well perceived and accepted, the ‘time factor’ related to the need to satisfy essential daily subsistence requirements played a significant role in determining participation in the screening. More well-adapted and meaningful Pro-ACD approaches could be implemented by improving the timing of the testing activites, and strengthening community participation and engagement to increase acceptability.

## Introduction

Globally, many malaria-affected countries are devoting more and more attention to the elimination of *Plasmodium falciparum* (Pf). In the Southeast Asian region in particular, elimination is high on the political agenda in order to prevent the many efforts towards malaria control being undone by the upcoming Pf resistance to artemisinin [1,2]. Malaria elimination is predicated by a rigorous surveillance system, which in turn requires the detection and real-time notification of each symptomatic malaria case, and a full three days of treatment and in-depth investigation [3].

However, passive surveillance alone cannot have a sustained exhaustive impact on malaria transmission. The bulk of infections, responsible for the existence of a consistent reservoir of parasites, does not only have a geographic focus (‘hotspots’), but is also clustered in demographic ‘hotpops’ [4,5], which are largely composed of asymptomatic individuals. It has been estimated that in low transmission areas, asymptomatic and sub-patent infections are the source of 20–50% of all human-mosquito transmissions [6,7]. Such ‘hotpops’ are characterised by a combination of malaria vulnerability, high rates of asymptomatic infections, and often a propensity for mobility, which makes them challenging to locate and intercept [5,8]. Strategies of pro-active case detection (Pro-ACD) build their approach on targeting asymptomatic, high-risk group ‘hotpops’, and may hold great promise in malaria elimination [4].

In the Greater Mekong Sub-region (GMS) the malaria ecosystem and transmission patterns are largely determined by forested areas and surrounding environments [9–11]. In recent years, Cambodia in particular has experienced a proliferation of land development projects, mainly in the agriculture sector, which has led to radical changes in the environment, and the destruction of huge forested areas. This has affected the regional malaria epidemiology and the general behaviour of local and migrant populations, who now have to move more frequently and over larger distances to reach the densely forested areas they depend on for their livelihood [12].

Apart from the classic ‘rural-to-rural migrant group’, defined as a small population of farmers leaving their rural communities to settle (sometimes permanently) in large-scale plantations or smaller farms, the Cambodian ‘mobile population’ has a very mixed profile, consisting of ‘cross-border migrants’ and ‘indigenous multiple residence system’ mobile groups [8]. The latter refers to local indigenous populations, whose daily or short-term movement is driven by subsistence requirements (i.e. sleeping at farms in the forest) and economic reasons such as staying overnight in the forest for logging [8,12,13]. Novel strategies to reach such highly mobile ‘hotpops’ are called for, and interventions such as pro-active case detection (Pro-ACD) have been proposed as a possible option [4,5]. So far, few studies in Cambodia and in the GMS have documented the pro-active targeting of mobile, asymptomatic subgroups [14]. Indeed, such approaches face the logistic challenge of mobility, as well as the limited propensity of asymptomatic carriers to actively seek testing for malaria and the limitations of the commonly available point-of-care diagnostic tests, which are unable to detect sub-microscopic parasitemia described among asymptomatic populations [6]. In response to these constraints and to the dearth of published evidence, Médecins Sans Frontières (MSF) conducted a Pro-ACD intervention in the northern province of Preah Vihear, Cambodia [15].

The Pro-ACD pilot study drew on assumptions about which populations are considered most at risk, and attempted to mobilise such populations for voluntary screening and treatment (VSAT) for malaria. It was designed as a mixed-methods study, with a quantitative arm aimed at exploring i) its feasibility, ii) extra yield of Pf infections detected and iii) convenience of defining both conventional (forest-goers) and non-conventional at-risk populations, while the qualitative arm (presented here) attempted to explore the understandings, expectations, perceptions and acceptability of Pro-ACD activities and the related VSAT mobilisation, as well as the motivations of the population to participate.

## Methods

### Study sites and description of the intervention

The study took place in the Chey Saen district of Preah Vihear province, Cambodia. Chey Saen is one of the seven administrative districts of Preah Vihear province, with an estimated population of 22,499 in 23 villages in 2014, as described elsewhere [16]. The area is served by a referral provincial hospital, three Health Centers and two Health Posts. Malaria transmission is seasonal, with a peak occurring in December-January at the end of the rainy season, and occurs mainly in the high risk areas of the forest and forest fringes [17], which mainly surround the east and south part of the district. In 2015, the incidence of symptomatic Pf infections in the district was 8.3/1.000 inhabitants/year [17].

Chey Saen has a passive surveillance system delivered through a network of village malaria workers (VMWs) and Health Centers. Since 2014, this network has been supported and trained by the international non-governmental organization MSF [17]. Each of the 23 villages has two VMWs who are able to diagnose malaria infections using rapid diagnostic tests (RDT) and provide treatment according to national guidelines. Since October 2015, VMWs have also routinely collected filter paper blood spots (Dry Blood Spot, DBS) for subsequent PCR diagnosis (through the Institut Pasteur du Cambodge; IPC), and alert MSF when a new case of Pf is identified. The first line treatment at the start of this pilot study was dihydroartemisinin-piperaquine (DHA–PPQ), which was changed to artesunate-mefloquine in February 2016 due to the high rate of Pf resistance to DHA-PPQ [18].

### Formative research

Before implementing the community mobilization for VSAT, formative research was conducted to gain insight and understanding into the social and economic context of the targeted villages. Data were collected through a questionnaire-based survey, meetings and observations in the villages. The socio-demographic characteristics and economic profile of the populations were identified and complemented with previously previous findings on health-seeking behavior and perceptions of malaria [19].

The formative research was instrumental in defining the essential role of formal and informal village leaders as gate-keepers for conveying messages to the population and assisting in gaining its trust. An important discovery during this formative stage was that it was not only men, but also women and children, who were moving frequently from villages to forest farms (plantations surrounded by forest) following a pattern of frequent, short-term mobility named ‘indigenous farming’ [8]. This data was instrumental in shaping the strategy of providing village-based screening sessions in the early morning and late evening, in order to be able to reach as many people as possible as they were coming back from the forest and plantations to the village.

### Qualitative study design

The strategy of the pilot Pro-ACD study, under the form of VSAT, revolved around the mobilization of specific high-risk groups, namely people who had spent at least one night in a forest and/or plantation and/or rice-field prior to the screening day. People with a previously reported history of malaria were also asked to participate in the screening activities. Those not meeting any of these criteria (the so-called ‘4 negatives’), but still presenting for screening, were also tested.

**Fig 1:**
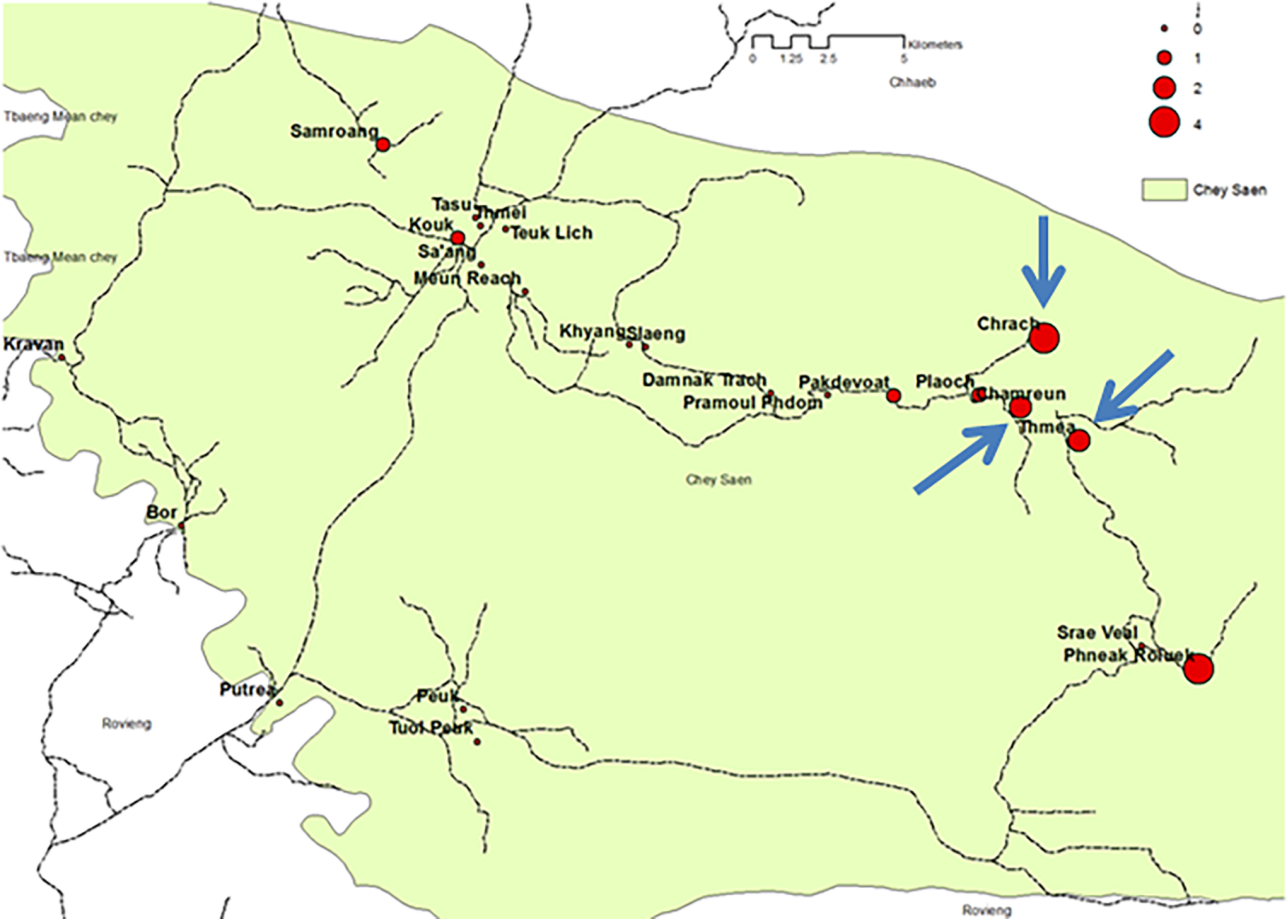
Prevalence survey in Chey Saen. Number and location of Pf infections and Pro-ACD villages indicated.

For the purposes of this study, three rural villages were chosen (Thmea, 2024 inhabitants; Chrach, 897 inhabitants; Chamreun 529 inhabitants) based on previous data showing high incidence and prevalence in 2014 (Fig 1) and from 2015, Pf infection [16,17]. Multiple testing rounds, each one lasting 1.5 days (with two evening sessions and one early morning session), were carried out in the villages on a monthly basis between December 2015 and March 2016. December and January are considered to be the peak of the transmission season, while February-March marks the beginning of the low transmission season [17]. In order to give people a greater opportunity to attend the screening, the testing activities spanned from early morning to late evening. This allowed people returning from occasional farming and forest activities to participate. No age limits were set.

In the two weeks prior to beginning the VSAT activities, health promotion (HP) sessions were conducted in each village. Specific meetings with previously identified formal and informal village leaders were organized to explain the new activity. Leaders were also invited to promote the Pro-ACD activities in their communities and to systematically attend all the screening sessions, in order to encourage participation. Several information and sensitisation sessions took place in the different villages, in which the concept of asymptomatic malaria was explained and the Pro-ACD testing services promoted. This concept is relatively new in the framework of malaria health promotion messages in Cambodia and in the GMS, with few noteworthy examples of introducing it among such communities being described only recently [20–22]. Nonetheless, in light of the lack of specific available HP messages in the Cambodian context, at the time of the VSAT implementation (December 2015), MSF had to create new material on this topic to help local villagers to understand more about the disease. The main HP messages were based on formative research conducted by an anthropologist during the implementation of the project, and centred on the idea that although asymptomatic malaria does not represent an immediate health problem, it could create future health issues if not adequately treated, potentially leading to economic loss for the individual and his/her family, at both individual and community levels.

**Fig 2:**
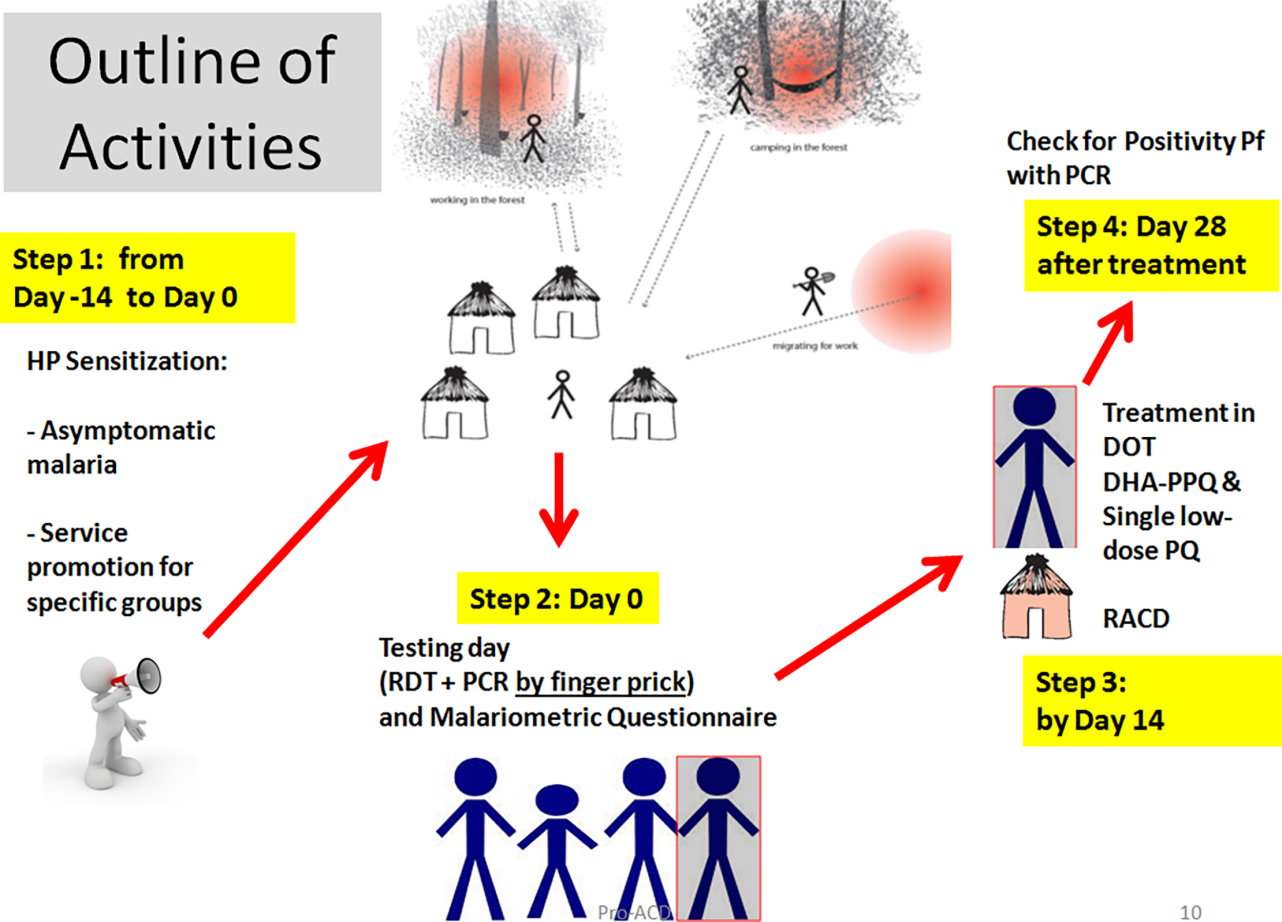
Outline of Pro-ACD activities.

In the two weeks prior to the testing day (Fig 2), the HP messages were conveyed verbally by three local HP officers (one per village) through village meetings and door to door sensitisation. The HP officers also announced the time and exact location of the testing activities using a megaphone and speakers as they toured the village. No incentives were given to the participants during testing activities. All participants, regardless of whether or not their test was positive or negative, were subsequently informed about the outcome of their results.

### Data collection

The qualitative study described here consisted of in-depth interviews (IDIs) and focus group discussions (FGDs). These were conducted using pre-developed topic guides based on observations collected by an anthropologist during the Pro-ACD activities, informal conversations with the team members and villagers about their experiences, and the information gathered from previous studies and assessments in the area.

Nine FGDs were conducted with groups of up to 10 community members. The FGD participants were purposively selected and recruited through the village leaders, VMWs and MSF HP officers, who were asked to identify villagers according to the following criteria: 1) people who participated in Pro-ACD and those who did not; 2) people who had *ever* spent at least one night in a forest, and/or plantation, and/or rice-field; 3) people aged 16–60 years, as this age category included most of the Pf cases identified through passive surveillance [17]. In each of the three villages, one FGD was conducted with villagers who did *not* participate in Pro-ACD; a second with Pro-ACD participants and a third with both non-participants and participants in Pro-ACD activities (mixed FGD). In total, three FGDs were held in each of the three villages. In total, 46 Pro-ACD participants and 40 Pro-ACD non-participants took part, of whom 47 were men and 39 women (Table 1).

**Table 1:** Overview of participants in qualitative study.

Type of data collection	IDIs		FGDs			
	Gatekeepers	VMW	Pro-ACD non-participants	Pro-ACD participants	Mixed	Pro-ACD and HP team
Number of FGDs/IDIs	6	3	3	3	3	1

Each FGD lasted between 90 minutes and two hours, and all were conducted in the local language of Khmer and facilitated by the PI and local Khmer-speaking research assistants. Each assistant had a specific task: one took notes and the other one translated to the PI as he asked questions. Both assistants probed responses for more detail where possible. All FGDs were audio-recorded with the verbal consent of the participants. Each evening, the team discussed the FGD and the notes they had taken, in order to make any necessary changes to data collection the following day.

In addition, nine IDIs were conducted with purposively selected VMWs, village chiefs, deputy chiefs and assistant chiefs from the three villages. These were done to ensure that perspectives from village leaders and from those involved in the provision of health care were included in the study and to allow people in positions of authority to have an individual voice and share their expertise and views. All IDIs were conducted in Khmer, and audio-recorded with the verbal consent of the participants.

Two Khmer-speaking research assistants were trained to conduct FGDs and IDIs and to take notes. One was already working in the project as a liason officer and was responsible for taking-notes during IDIs and FGDs and helping the team during the analysis of the data. He was able to highlight any discrepancies between the interviewees’ answers and the history of the project. The principal investigator, a trained anthropologist, observed the activities and answered questions from participants about the study. At the end of each FGD and IDI, the research team discussed the information collected, in order to adjust and refine the next questions to be asked to the villagers.

### Transcription and data analysis

When data collection was complete, all audio-recordings were translated from Khmer to English and transcribed before analysis. This was followed by a second process of review to ensure the quality of the data: English transcripts were read by the study team, compared with the Khmer audio-recording and any differences were discussed and discrepancies corrected. Data analysis was conducted manually by the PI and discussed with the rest of the team.

Data analysis centred on the identification of the villagers’ perspectives and views on the following topics: a) knowledge (signs, timing of transmission, basic prevention tools–use of mosquito net and long sleeved clothing) and perceptions of malaria risk; b) knowledge of the Pro-ACD activities and understanding of the HP messages targeting high-risk groups; c) expectations and acceptability of Pro-ACD and d) participation in Pro-ACD activities. The analysis of the qualitative data was an inductive iterative process, where data was repeatedly explored and interpreted by the PI and discussed within the team [23]. The transcript from each FGD (Pro-ACD non participants, Pro-ACD participants, and mixed groups) was coded in sub-texts consisting of the answers of the interviewees, which were revised and categorized around the themes of ‘expectations’ and ‘acceptability of the Pro-ACD’, as these topics emerged as the main drivers of discussion after the first reading and analysis of the data. This analysis was supported by available literature and enhanced by the daily discussion of the results within the team.

This qualitative study was approved by the Cambodian National Ethics Committee on Health Research (NECHR, approved 24th June 2013, number 0094NECHR). The qualitative research team also followed the Code of Ethics of the American Anthropological Association [24]. Before participating in the FGDs and IDIs, participants were informed about the objectives of the project, the topic and type of questions as well as their right to withdraw their participation at any time. All participants gave their verbal consent before participating.

### Characteristics of research participants

**Fig 3:**
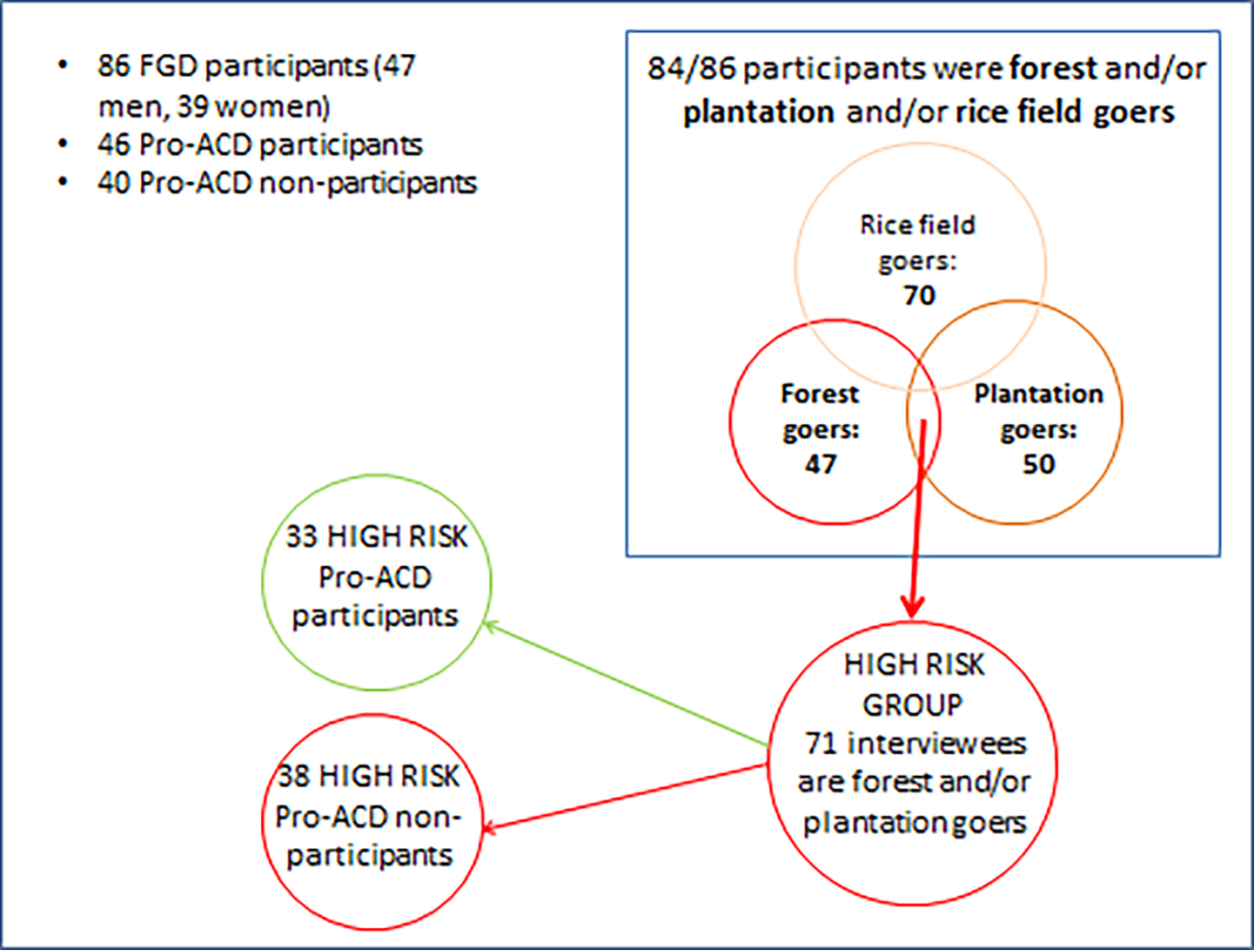
Characteristics of FGD participants.

Out of the 86 FGD participants, 71 reported to have spent a night specifically in the forest and/or plantation. From previous studies in Cambodia [9], it is known that these behavioural patterns are considered to confer high risk for developing Pf infection. Interestingly, among these 71 ‘high risk’ people, a balance between Pro-ACD participants (33, 46.5%) and non-Pro-ACD participants (38, 53.5%) was achieved (Fig 3).

## Results

### Villagers’ knowledge of Pro-ACD activities: Understanding of health promotion messages

As reported in routinely collected, internal project data, the Pro-ACD screening in the three villages reached a general coverage of 54% after three rounds. Approximately 22% of the participants (622 out of the 2802 total screened) did not fall within the mobilisation criteria for the target groups (the group known as the ‘4 negatives’).

One of the themes we explored was how HP messages were understood by the local population, and whether or not people had received the messages that were disseminated by the HP team.

The majority of the participants reported that they were first asked if ‘*we used to go to the forest or rice field*, *and if we sleep there*, *in the mosquito net or not’*. Another man said ‘*[MSF HP officer] said she wanted me to have a blood test for malaria since I had been in the forest*’ (male Pro-ACD participant from Chrach).

Those who did not participate in the Pro-ACD activities confirmed that they had heard the announcement about the screening activities, yet still did not participate. A male FGD participant in Thmea reported ‘*they* [HP team] *came to tell us about the blood test campaign visit to the village*, *but I never joined it’*, and a young man from Chrach added ‘*some doctors came door to door to tell us that “tomorrow there will be a blood test campaign in our village”‘*.

Villagers clearly expressed the sense that most of the people in the villages received the message from the ‘*MSF doctors’*–as they often called the HP team—through door-to-door visits and megaphone announcements. In Thmea, a male FGD participant said:

They travelled by the motorbike through the village to tell everyone by megaphone, “*Please everyone*, *go to have blood tested for malaria”*.

This was confirmed in an interview by the VMW of Thmea, according to whom the majority of the villagers *‘understood the message because almost everybody has had malaria’*.

In Chrach village, during a discussion about the ‘forest environment’ being the classic, risky place for getting malaria infection, a woman intervened by pointing out that she was also at risk because ‘*we go to the plantation*, *we go as a family*’. In an FGD in Chamreun, both participants and non-participants from the Pro-ACD activities identified the people most at risk as ‘*the group who go to the forest*, *plantation or farm without sleeping in the mosquito net*, *regardless of the fact that they are burning fire*’.

### Malaria knowledge, prevention and perceptions of risk

As a result of the ongoing exposure of the villagers to the HP messages before the implementation of the VSAT, the majority of the villagers knew about the disease and malaria-related symptoms (fever, headache, generalised pain, and gastro-intestinal problems). Some Pro-ACD participants gave more precise details about the disease and the treatment required. In the FGD with Pro-ACD participants from Chamreun, a woman said ‘*malaria is caused by female Anopheles mosquitoes*’ and ‘*malaria can cause the rupture of blood cells*’. Occasionally, people from the FGDs with Pro-ACD participants, including a female participant from Chrach, wrongly reported that malaria can relapse *‘because of the food eaten earlier*’. Among the non-participants in the Pro-ACD testing, a man said ‘[we can have] a *relapse of malaria because we do heavy work*’, while another man replied ‘y*es*, [if] *we do heavy work*, *malaria will re-occur* […] *To definitely get cured*, *we must finish all the tablets provided by doctor and follow the instructions as well*.’

The importance of treatment adherence recurred on numerous occasions during the various FGDs. Participants and non-participants in the Pro-ACD activities understood the implicit risks related to not completing treatment. In Chrach village, a woman stated that some villagers had a ‘*malaria relapse*’ because they didn’t get ‘*enough doses of medicine’*. Another female participant said that:

Patients are supposed to take a 3-day dose of medicine, but they only consume medicine for two days; disease is not definitely killed…that’s why there is the relapse. To get cured, the medicine should be taken completely, daily and regularly.

The majority of the participants identified forests and plantations as the places where there is the highest risk of contracting malaria, but the overall perception of the risk appeared to extend beyond these geographical contexts and the ‘4 negatives’, by also including the village environment. A female participant from an FGD with Pro-ACD participants in Thmea reframed the issue as a ‘*lack of sanitatio*n’, because of the presence of stagnant water in the village, which ‘*attracts a lot of mosquitoes*’. A man from a FGD of those who did not participate in the Pro-ACD in Chamreun expressed the same concept, identifying the people at risk as ‘*the ones* [villagers] *whose houses are not properly clean*. *For example*, *there are water dumps or open cans on the ground; when it rains*, *this is going to have more mosquitoes*’.

When considering which groups of people would be most at risk of getting malaria, the majority of the FGD interviewees (in particular the Pro-ACD participants) indicated those with certain ‘negative’ habits and behaviours, especially in the forest, by pointing to ‘*the ones who don’t like to sleep inside the mosquito net’*, *‘the ones who don’t like to wear clothes’* and the ones who ‘*do not pay attention to the announcement by the doctor*’.

### Perceptions, acceptability and participation in Pro-ACD activities

Participants such as these perceived the screening as an opportunity to ‘*participate in the blood test campaign in order to protect our health and our family from any disease*’ so that they could *‘have good health and get away from malaria*’.

Two constitutive elements appear to determine the decision to take part in the Pro-ACD activities: convenient timing of the announcement and freedom from working duties. The timing of the announcement played a pivotal role in people’s decision-making processes about participation. One FGD participant said ‘*we will participate as long as we have the information*’, followed by the clarification ‘*at least three days ahead*’.

Moreover, people from an FGD with Pro-ACD non-participants clearly stated that their attendance depended upon their availability. A man from Thmea said ‘*I did not participate because I was too busy…I didn’t come…I went to the forest at that time*’. In Chamreun village, another man stated that he could not take part in any of the screening sessions because ‘*they told us the date and place to have blood test*, *but unfortunately I was busy on the same day*’.

In summary, from the analysis of the FGDs, there was no indication of either a refusal or rejection of participating in the Pro-ACD activities. Instead, it was evident that the vilagers did not attend the testing activities mainly because they had to choose between working in the field or staying in the village for the test. In this regard, a woman said ‘*I never intended to escape* [not be tested]*; I knew when this doctor was coming*. […] *I could not join because I had to work for pay*’.

The concern around the lack of essential financial resources was identified as the central issue in the villagers’ lives. In Thmea village, a man said, ‘*I know that I might be infected with malaria when I go to the forest*, *but I still go*’, while another man supported him by saying ‘*we still persistently go to that forest* […] *because if we do not go our family will have nothing to eat*’.

FGD participants who took part in the screening were not in opposition to those who did not. Indeed, the former decided to be tested because, as a woman from Chrach said, ‘*we will spend a lot of money to go to a doctor if we have malaria*’. Another man who participated in the Pro-ACD activities reiterated this idea:

It [malaria] affects the whole family in terms of money and time. For example, I can earn 20,000 riels [4000 riels equals 1USD] per day, so if my wife is sick, and we spend 40,000 riels per day for treatment; how much will it cost if I have to spend 40,000 riels for 10 days for her treatment? It’s going to be a large amount of money lost.

Furthermore, the acceptablity of the Pro-ACD screening was related to both the simplicity and reliability of the procedure (a screening test and then treatment if the result was positive) and the perceived efficacy of the drug used. For example, a male from Chrach who was screened and previously detected with malaria infection stated that ‘*at the third campaign*, *I came again for a blood test*, *but fortunately*, *I was found malaria negative*’, while another man from Chamreun said ‘*I am so happy to receive malaria blood testing*. *I strongly hope the doctors will do it every year and forever so that the number of malaria cases will be decreased*’.

Moreover, the village chief of Chamreun emphasized the lack of harm related to the malaria screening activity. He ‘*looked at the MSF team during the VSAT procedure and verified how the nurses were caring for the patients*: *first giving an appointment and then giving medicine and observing the sick people taking it*’. He then reported that he felt ‘*reassured and promoted the activity*, *trying to explain it to the villagers*’.

## Discussion

From the qualitative analysis of the pilot of Pro-ACD the following elements emerge: a) the HP messages were well received, although understanding of the concepts of ‘asymptomatic malaria’ and transmission dynamics were difficult to grasp in their entirety; b) there was a consistently positive opinion about the Pro-ACD intervention. Acceptability was high, and screening activities were perceived favourably. Pro-ACD intervention was gauged by the local population to be effective, inexpensive, reliable and readily available; c) no concerns regarding testing or treatment were identified during the analysis of IDIs and FGDs; d) lack of time to attend the VSAT and, to a lesser extent, physical absence (both linked to work-related activities), were the main reasons for non-participation in the Pro-ACD screening.

In the village context, where life depends on subsistence farming, day-to-day resources are limited and uncertain. The immediate needs are i) domestic (daily supply of food and water), ii) work-related (provision of fuel for machinery) and iii) social (participation in local ceremonies). Finding solutions to earn essential money for basic daily goods is the most common concern for the villagers. Their pattern of saving does not provide enough surplus for possible emergency scenarios, such as being unable to work because of illness.

At first glance, and as has been documented elsewhere [25], the economic element linked to working activities appeared to be the most important reason for non-participation in Pro-ACD activities, and a lack of both free time and physical availability due to work commitments played a pivotal role for both participants and non-participants in deciding whether or not to be screened. However, for the Pro-ACD participants, the same economic issue was the main driver for attending the screening. They wanted to be screened to avoid developing malaria, thus avoiding future economic insecurity related to treatment costs and loss of income.

It is reasonable to assume that the innovative introduction of the Pro-ACD screening activity (provided for free, within the community and able to identify and treat malaria cases before their overt clinical manifestation) was positively welcomed by the majority of the population as a protective and preventive tool capable to avert the detrimental vicious cycle of ‘poverty-malaria’ [26]. This is particularly observed in rural contexts such as this one, where the population discuss placing less importance on the risk of getting the disease when compared to the risk of poverty, as they repeatedly enter zones where they are at risk of contracting malaria [27].

During FGDs, participants demonstrated that they had received and understood the main topics of the HP messages (malaria symptoms, transmission and prevention), as well as the announcement of the screening activities. Service promotion reached most of the people in the targeted villages: only a minority of them were not aware of the existence of the activities because they were in the forest or plantation at the time of the village information sessions. Based on this information, it is evident that a specific group was asked to participate in the testing and that villagers clearly understood this request.

While the majority of the villagers appeared to have understood the VSAT selection criteria for mobilisation (namely, previously spending a night in the forest, plantation or rice-field and/or having a past history of malaria), some who did not have any of those criteria still decided to participate in the Pro-ACD. The ‘4 negatives’ group attendance rate (22% of the total participants, according to the quantitative evaluation of the pilot Pro-ACD study) represents a non-negligible proportion, which deserves further consideration.

Possible explanations of this phenomenon could be related to the fact that not all of the population completely understood or interpreted the HP messages, and that the ‘4 negatives’ villagers stated that they felt at risk of malaria because of the ubiquitous presence of mosquitoes inside the village. This fact, grounded in the generalised knowledge that malaria is transmitted by mosquitoes, was sufficient for the villagers to disregard the messages that forests and plantations are the only risk factors for malaria. Instead, people adapted the HP messages received (*malaria is transmitted by mosquitoes*) with their experiences and perception of the reality (*mosquitoes are also present at village level*).

The topic of prevention and the concern for observing proper behaviours in forest, plantation and ricefield environments frequently occurred in the discourse of the Pro-ACD participants during the FGDs. Their reported beliefs suggest that it is more pragmatic and meaningful to implement preventive practices rather than avoiding high-risk sites linked to their main economic activity to decrease the risk of mosquito bites and then the risk of becoming sick from malaria. These findings resonate well with those from recent qualitative analyses on mass drug administration (MDA) in Cambodia and Vietnam: in those studies, villagers highlighted the importance of implemeting protective measures, as they perceived malaria to be a concrete threat [22, 28].

Yet, as more complex concepts around malaria such as relapse/recrudescence and symptomatic/asymptomatic were given to the villagers, difficulties in elaborating and understanding them clearly emerged during the FGDs. This was not surprisingly, as it has been already reported by other study groups addressing community mobilization for MDA [20, 22, 29]. Suggestions proposed by some of them included trying to iteratively modify the specific messages based on continuous formative research enabling the adaptation of messages to the local context [20] and attempting to simplify the message by focusing the village’s attention on the main concept of ‘malaria elimination’ [22]. In this regard, we can reflect on whether or not more effort should be geared towards building better strategies for future HP activities, such as the delivery of simple and culturally sensitive messages, conveyed in user-friendly ways (such as dividing complex ideas in several messages and separate sessions). Interpersonal communication, in this context, could also be considered for specific groups of people such as forest and plantation workers [30].

The idea of creating target groups of forest- and plantation-goers for targetted health promotion sessions, providing them with specific information on avoidable work-related habits and risky behaviours in specific high-risk environments for malaria, could in turn prove effective in generating the self-identification of risk exposure, thus boosting their participation in future VSAT activities.

Both the Pro-ACD participants and non-participants appreciated the screening, its aim and the way it was carried out, with non-participants stating their wish to have future opportunities to be tested.

There are several strengths and limitations to this study. Firstly, those who did not participate in the Pro-ACD screening may have been reluctant to share their reasons for non-participation with the team, thus introducing a potential bias into the findings. In addition, the presence of a translator may have introduced an additional element of bias, as well as leading to possible misinterpretations. This bias was reduced by ensuring that all FGD and IDI data were discussed with the team during analysis and any discrepancies clarified. However, despite these limitations, this study offers a unique qualitative insight into Pro-ACD screening, under the form of a VSAT approach, in a rural Cambodian community. Another strength lies in the choice of methodology, and the involvement of villagers who chose to participate, chose not to participate and who were involved in the decision making process about the VSAT implementation.

## Conclusion

In view of these positive findings, a meaningful expansion of the Pro-ACD screening can be proposed in the Cambodian context where MSF works. Certainly, there is a need to carefully consider the work burden of the forest and plantation workers, who are often away from the village during daytime and for specific periods of the year. Their main priority is work [27], not screening for malaria, especially if they do not have any symptoms.

Participation could also be improved by the coordinated implementation of a community participatory model [31–33], that adds to the already existing efforts built around the i) involvement of local formal and informal authorities, ii) implementation of formative research, iii) sharing the results with the community [20]. In this sense, the positive experience of forest or plantation-goers who benefitted from the screeningcould be harnessed by engaging them as voluntary ‘role models’, able to encourage more at-risk groups to attend future screenings through discussing their own experiences with others.

To be successful, these strategies should be integrated with a contextually adapted approach towards the community, which could consolidate and simplify the main messages around malaria prevention and elimination, while addressing any misunderstandings in order to unleash the potential of the community engagement element as an instrumental factor for achieving elimination of malaria at village level in Cambodia.
